# Cancer of the Bladder—Puzzling Case

**Published:** 1831

**Authors:** 


Cancer of the Bladder — puzzling Case.

[Hôpital St. Louis.]

A man named Rossignot, aged 65 years, entered the hospital on the 13th Jan. 1831, saying he had been afflicted with rheumatism for a long time. His complexion was delicate, his limbs slender, and his features shrunk—in fact, he was greatly emaciated. He complained of a burning heat internally, and had much thirst, with constipation of the bowels, but little or no tenderness on pressure of the abdomen. He had severe pains in the loins, and indeed in both of the lower extremities. He had incontinence of urine, and his body and clothes smelt strongly of that excretion. This incontinence had existed for eighteen months, with some periods of retention of the same, the urine being very turbid, and sometimes mixed with blood. M. Biett conceived that there was some organic disease of the urinary organs. The pains in his loins increased rather than diminished; and on the night of the 16th of the same month, he was seized with hæmaturia; and the next day, in the midst of interrogations, he suddenly expired. The reporter, M. Berard, here asks what medical man, after observing the above symptoms, would hesitate to pronounce the disease Nephritis Calculosa? This opinion was that formed by M. Biett, and participated in by M. Berard; yet they were both mistaken.

On dissection, the kidneys were free from disease. The ureters were as large as the jejunum. The bladder filled a great part of the inferior pelvis, and was knotty on its surface, its cavity almost obliterated, and its parietes thickened enormously by a malignant fungoid disease. A cauliflower excrescence, of a cancerous character projected from the bladder towards the inguinal ring; and another penetrated through the great ischiatic notch, compressing and blending with the great sciatic nerve. A third excrescence ascended from the fundus of the bladder towards the lumbar vertebræ, compressing the ureters, and obstructing the discharge of urine into the bladder. These post mortem facts explained all the vital phenomena, though it would not have been easy to predicate the real nature of the malady during life.—*Revue Medicale*.

